# IRE1α Inhibitors as a Promising Therapeutic Strategy in Blood Malignancies

**DOI:** 10.3390/cancers14102526

**Published:** 2022-05-20

**Authors:** Wojciech Wiese, Natalia Siwecka, Adam Wawrzynkiewicz, Wioletta Rozpędek-Kamińska, Ewa Kucharska, Ireneusz Majsterek

**Affiliations:** 1 Department of Clinical Chemistry and Biochemistry, Medical University of Lodz, 90-419 Lodz, Poland; wojciech.wiese@stud.umed.lodz.pl (W.W.); natalia.siwecka@stud.umed.lodz.pl (N.S.); adam.wawrzynkiewicz@stud.umed.lodz.pl (A.W.); wioletta.rozpedek@umed.lodz.pl (W.R.-K.); 2 Department of Gerontology, Geriatrics and Social Work, Jesuit University Ignatianum, 31-501 Krakow, Poland; ewa.kucharska@ignatianum.edu.pl

**Keywords:** endoplasmic reticulum stress, unfolded protein response, inositol-requiring enzyme 1 alpha (IRE1α), X-box-binding protein 1 (XBP1), blood cancer, leukemia, lymphoma, multiple myeloma

## Abstract

**Simple Summary:**

Blood malignancies account for 6.9% of all cancer deaths. Inositol-requiring enzyme 1 alpha (IRE1α), a part of the unfolded protein response (UPR), has been shown to be pivotal for cancer cell development and progression, including blood cancers. Furthermore, IRE1α levels are often elevated in blood cancer cells, and they correspond with cell survival, response to treatment, and prognosis. The aim of our study is to summarize the current knowledge on IRE1α in blood cancers and to evaluate the potential utility of IRE1α inhibitors in the treatment of blood malignancies. The introduction of new therapies based on IRE1α inhibition may increase treatment efficacy and reduce the side effects of blood cancer therapy.

**Abstract:**

Synthesis, folding, and structural maturation of proteins occur in the endoplasmic reticulum (ER). Accumulation of misfolded or unfolded proteins in the ER lumen contributes to the induction of ER stress and activation of the unfolded protein response (UPR) signaling pathway. Under ER stress, the UPR tries to maintain cellular homeostasis through different pathways, including the inositol-requiring enzyme 1 alpha (IRE1α)-dependent ones. IRE1α is located in an ER membrane, and it is evolutionarily the oldest UPR sensor. Activation of IRE1α via ER stress triggers the formation of the spliced form of XBP1 (XBP1s), which has been linked to a pro-survival effect in cancer cells. The role of IRE1α is critical for blood cancer cells, and it was found that the levels of IRE1α and XBP1s are elevated in various hematological malignancies. This review paper is focused on summarizing the latest knowledge about the role of IRE1α and on the assessment of the potential utility of IRE1α inhibitors in blood cancers.

## 1. Introduction

Blood cancers belong to a heterogeneous group of tumors of the bone marrow, blood morphotic elements, and lymphoid organs. In general, such tumors comprise three major subgroups: leukemias (acute lymphocytic leukemia (ALL), acute myeloid leukemia (AML), chronic lymphocytic leukemia (CLL), or chronic myeloid leukemia (CML)), lymphomas (grouped into Hodgkin lymphomas (HL) and non-Hodgkin lymphomas (NHL)). and multiple myeloma (MM), depending on the derivation and histological features of the affected cells. Blood malignancies account for 6.2% of all cancer cases and 6.9% of cancer deaths—every year, there are more than 1.27 million cases and 700,000 deaths due to blood cancer [1,2]. The prevalence of hematological malignancies is closely related to age, and it significantly varies for different types of malignancies. Overall, the development of blood malignancies is linked to genetics and several environmental factors, which include exposure to air pollution, ionizing radiation, chemicals, and smoking [3]. Blood cancers also frequently coexist with autoimmune disorders such as rheumatoid arthritis, systemic lupus erythematosus, or celiac disease [4,5,6]. Notably, lymphomas such as HL, Burkitt lymphoma (BL), and diffuse large B cell lymphoma (DLBCL) are often associated with Epstein–Barr virus (EBV) infection [7,8].

Blood cancer treatment outcomes vary significantly—from successfully treatable HL to resistant and poorly treatable AML. Current treatment methods such as chemotherapy and radiation therapy for blood cancers, apart from HL, are not fully effective, and they also cause multiple side effects such as heart failure [9], infertility [10], and even second malignant neoplasms [11]. On this account, it is desired to keep looking for more effective and less toxic treatment options. Herein, we would like to focus on new opportunities in the treatment of blood cancers, that can be provided by the specific inositol-requiring enzyme 1α (IRE1α) inhibitors. IRE1α is a transmembrane endoplasmic reticulum (ER) membrane receptor that serves as an ER stress sensor, and it is also involved in the maintenance of cell proteostasis by its dual kinase/endoribonuclease enzymatic function [12]. Increased activity of IRE1α has been linked to numerous hematological malignancies, including AML, pre-B ALL, CML, and MM [13,14,15,16]. In the present study, we present the current knowledge we gathered on distinct functionalities of IRE1α in blood cancer cells and discuss the potential utility of IRE1α inhibitors in the treatment of blood malignancies.

## 2. IRE1α Activation upon Endoplasmic Reticulum (ER) Stress Conditions

ER plays a major role in the synthesis, folding, and structural maturation of more than 30% of all proteins produced in the cell [17]. Newly synthesized polypeptides are folded and modified in the ER lumen to obtain their proper tertiary structure and, thus, function. Impairment of these processes leads to ER stress as a result of the accumulation of misfolded or unfolded proteins within the ER lumen [18], and such conditions trigger activation of the unfolded protein response (UPR) signaling pathway.

In general, UPR comprises three proteins—inositol-requiring enzyme 1 (IRE1), activating transcription factor 6 (ATF6), and PKR-like ER kinase (PERK) [19,20,21]. IRE1 is evolutionarily the oldest UPR sensor, as it was first identified in yeast *Saccharomyces cerevisiae*. For this reason, it is the best-known of all UPR-related proteins. In humans, IRE1 is encoded by the *Ern1* gene [22]. Mammals encode two IRE1 isoforms—IRE1α and IRE1β, of which the IRE1α isoform is more common in human cells [23]. IRE1α serves as a kinase and endoribonuclease that is located in the membrane of the ER. ER stress initiates IRE1 activity by modifying multiple chaperones that are mainly associated with pro-survival pathways. During ER stress, IRE1α undergoes oligomerization and activation when it is released from GRP78/BiP chaperone [24]. Recently, it has been suggested that the number of assembled IRE1 molecules results in distinct functionalities—the regulated IRE1-dependent decay (RIDD) is performed by IRE1 monomers/dimers, and it does not require oligomerization, whereas the XBP1 splicing reaction occurs upon formation of at least IRE1 tetramers [25,26]. Alternatively, approximately 5% of IRE1 molecules in a cell upon ER stress form clusters. These clusters have dynamic and complex structures adjacent to the ER membrane. The exact role of IRE1 clusters is unknown: They may be involved in splicing, storing excess IRE1, or be involved in the incorporation of additional signaling molecules that convey information independently of the RNase activity of IRE1 [27]. When oligomerized, the kinase domains of IRE1α subunits undergo trans-autophosphorylation, which induces activation of the kinase and RNase domains [24].

## 3. Different Outputs of IRE1α Activity upon ER Stress Conditions

Upon IRE1α activation, the RNase domain triggers the unconventional splicing of *XBP1* mRNA. As a result of this process, the homeostatic transcription factor XBP1s is produced [28]. XBP1s protein is a transcription factor that promotes cell survival via upregulation of pro-survival pathways, such as Myc proto-oncogene [29,30]. XBP1s is also involved in the induction of the expression of many other essential proteins such as granzyme B [28], interleukin 6 (Il-6) [31], and NK group 2 member D (NKG2D) ligand major histocompatibility complex class I polypeptide-related sequence A/B [32]. Importantly, XBP1s is also required for plasma cell differentiation [33]. High expression of XBP1s protein correlates with poor prognosis in several types of cancer, e.g., glioblastoma [34], triple-negative breast cancer (TNBC) [35], and pre-B ALL [14].

During ER stress or otherwise triggered autophosphorylation, the IRE1α RNase domain also causes endonucleolytic degradation of many mRNAs located in the ER that are structurally similar to *XBP1* mRNA [36]. As many degraded mRNAs encode for chaperones, these early events may terminate in apoptosis. This process is known as RIDD [37], as discovered in 2006 by Hollien and Weissmann in Drosophila melanogaster [38]. RIDD is constitutively active under basal conditions when there is no IRE1 signaling activated or ER stress. During elevated IRE1 activation and enhanced intensity or duration of ER stress, RIDD activity gradually increases [39,40,41]. In contrast, ER stress-induced *XBP1* mRNA splicing shows no correlation with the intensity and duration of ER stress. After exceeding a specific threshold, upon prolonged and unmitigated ER stress, RIDD eventually becomes cytotoxic. During this terminal UPR, the cytoprotective XBP1 mRNA splicing decreases while RIDD activity increases [42]. The pool of mRNAs degraded by RIDD activity depends on the cell type and, in general, is specific for mRNAs that encode proteins of the secretory pathway that are prone to misfold, which is the case for MM pathology [36,41,43,44,45,46].

The other specific activity of IRE1α, triggered by its kinase domain, is the activation of pro-apoptotic mechanisms. It has been shown that TNF receptor-associated factor 2 (TRAF2) can be directly activated by IRE1α. TRAF2 is known to activate apoptosis signal-regulating kinase 1/MAP3K5 (ASK1) and its downstream target, c-Jun N terminal kinase 1 (JNK/MAPK8/SAPK1) [23,47]. Such TRAF2/ASK1/JNK-dependent activity of IRE1 precedes the unconventional XBP1 splicing [48]. Furthermore, TRAF2 activates BCL2-associated X (Bax)/B-cell lymphoma 2 (BCL2)-regulated Ca^2+^ release from the ER [49]. The BCL2 family of proteins controls and regulates the intrinsic or mitochondrial apoptotic pathway [50]. Moreover, the inflammatory environment is promoted by the activation of IRE1, which, when activated, stimulates the JNK pathway. The JNK-dependent signaling promotes the secretion of pro-inflammatory cytokines, and it initially regulates cell survival by inducing the expression of antiapoptotic genes [47]. It has been hypothesized that JNK signaling becomes pro-apoptotic after 12 h of activation, as a late response to ER stress conditions [48]. Besides apoptotic pathways being activated and regulated by JNK, JNK may also participate in necrosis, as a response to ER stressors [51].

**Figure 1:**
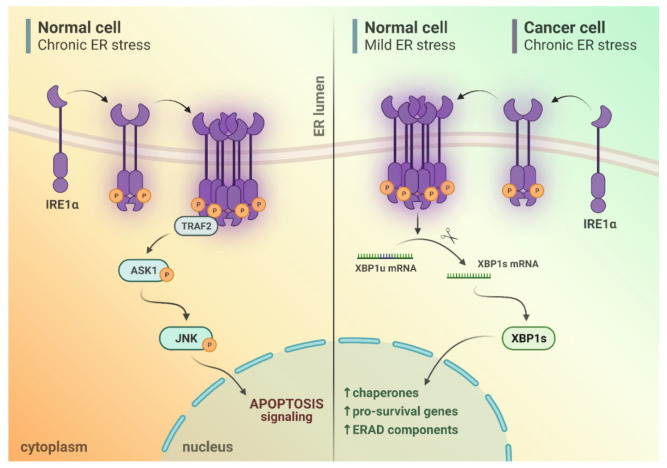
Different outputs of IRE1α activation depending on ER stress duration and type of affected cell: In non-cancerous cells, mild ER stress induces cytoprotective response via splicing of XBP1 mRNA, whilst chronic ER stress switches IRE1α activity rather toward induction of TRAF2/ASK1/JNK pro-apoptotic pathway. Cancer cells which exhibit chronic ER stress conditions due to their specific microenvironment are able to omit UPR-induced cell death. The intensity of IRE1α-dependent XBP1 splicing in these cells is significantly increased.

When the ER is under stress conditions, the UPR is activated in an attempt to maintain cellular homeostasis. When maintenance of homeostasis fails, the UPR activation leads to cell apoptosis. In hematopoietic cells, including MM, lymphoma, and acute T-cell leukemia cell lines, ER stress leads to caspase-induced cleavage of IRE1, generating a stable IRE1 fragment consisting of an ER-lumenal domain and a transmembrane segment. This cleavage disconnects the stress sensing and signaling fragments, resulting in a decrease in IRE1 activity [52]. Under mild ER stress, IRE1 signaling of UPR plays a role in pro-survival via activation of the XBP1 branch [53,54,55,56], and partly via RIDD [57]. In contrast, continuous and chronic stress shifts UPR signaling toward pro-apoptotic activity [54,55,58], which is apparently RIDD- and JNK-dependent [59,60]. Cancer cells that are chronically exposed to multiple environmental stressors are known to overexpress IRE1 and XBP1 factors so as to shift the balance from pro-apoptotic toward pro-survival downstream pathways [61] (Figure 1).

## 4. IRE1α in Blood Malignancies

### 4.1. Chronic Myelogenous Leukemia

Recently, it was found that a new generation proteasome inhibitor oprozomib leads CML cells towards apoptosis through a synergistic effect of calcium leakage and phosphorylation of PERK and IRE1α [15].

CML cells demonstrate ER stress conditions, and as a major ER stress sensor, IRE1α supports CML cell survival. Inhibition of IRE1α or NOD-like receptor pyrin-domain-containing 1 (NLRP1) decreased proliferation and increased apoptosis of CML cells, whereas overexpression of IRE1α- or NLRP1-encoding genes showed opposite effects. Knockdown of the IRE1α–NLRP1 pathway made CML cells vulnerable to apoptosis induced by imatinib. Primary cells obtained from CML patients exhibited increased expression of IRE1α and activated NLRP1 inflammasome, whereas inhibition of IRE1α or NLRP1 led to reduced proliferation and increased apoptosis of primary CML cells. Therefore, the IRE1α–CREB–NLRP1 pathway promotes CML progression and resistance to imatinib [62].

Hematopoietic stem cells (HSCs) are protected from ER-stress-induced apoptosis by adaptive signaling of the UPR, IRE1α–XBP1. Blockage of IRE1α results in decreased reconstitution of HSCs. Under ER stress conditions, IRE1α–XBP1 is activated by N-RasG12D through MEK-GSK3β to promote HSCs survival. Knockdown of IRE1α–XBP inhibited N-RasG12D-induced survival during ER stress and reduced the competitive advantage of NrasG12D HSCs in transplant recipients [63].

### 4.2. Chronic Lymphocytic Leukemia

Myc activates the pro-survival IRE1α–XBP1 pathway in CLL [64]. Moreover, deficiency of XBP1 reduces the progression of CLL in a mouse model. XBP1 deficiency resulted in impaired BCR signaling and increased surface expression of the sphingosine-1-phosphate receptor. Inhibition of the ER transmembrane receptor IRE1, required for XBP1 expression by a selective IRE1 RNase inhibitor B-I09 resulted in XBP1 deficiency accompanied by increased IRE1 expression and impaired BCR signaling. Treatment with B-I09 in a mouse model of CLL inhibited leukemia progression through induction of apoptosis and did not cause systemic toxicity [65].

### 4.3. Acute Myeloid Leukemia

Expression levels of XBP1 and XBP1s are elevated in AML. *XBP1s* mRNA expression is markedly higher in both bone marrow and peripheral blood samples from AML patients than that in samples from healthy individuals [66]. In another study, XBP1s formation was reported in 16.2% (17 of 105) of AML patients, which suggests that the IRE1α-dependent signaling of the UPR is activated in some AML cases. Additionally, patients with activated UPR were characterized by a better prognosis [67]. In mouse models of AML, ER stress can pass from AML cells to bone marrow cells. This results in increased UPR activation, accelerating osteolytic differentiation of mesenchymal stem cells. This phenomenon is believed to be the origin of chemoresistance [68]. IRE1α inhibition in AML induced caspase-dependent apoptosis and cell-cycle arrest in the G1 phase. Mechanistically, this occurred partly through the upregulation of BCL2 family proteins, proteins that control the G1 phase, and chaperones [13]. This suggests that AML cell survival is IRE1α-dependent. Jun proto-oncogene (JUN) is a regulator of the UPR in AML. JUN during ER stress induces XBP1 and ATF4. Induction of mentioned UPR effectors, in turn, enables AML cell survival during stressful conditions. Hence, JUN and UPR may become potential therapeutic targets in AML [69].

IRE1a is also activated in one aggressive subtype of AML—mast cell leukemia (MCL). Inhibition of IRE1α attenuated proliferation and induced apoptosis in MCL cells. This suggests that IRE1α may be a prospective target against MCL [70].

### 4.4. Acute Lymphoblastic Leukemia

Pre-B ALL cells are extremely susceptible to ER stress. In pre-B ALL, promoter regions of the *Ern1* gene have low levels of CpG methylation [14], whereas expression of the entire *Ern1* gene is downregulated in B-ALL [71]. High XBP1 levels in pre-B ALL are related to poorer patient prognosis. The UPR and its effector XBP1 are identified as new targets to overcome drug resistance in pre-B ALL [14]. Notably, nuclear expression of XBP1 occurs in reactive plasma cells and also in B cells Irf-4^+^/Bcl-6^−^/Pax-5^−^ in bright zones of reactive nucleated centers that likely represent cells involved in plasmacytic differentiation [72]. In T-ALL, NOTCH3 silencing results in a BiP-dependent inactivation of IRE1α. This inactivation of IRE1α under stress conditions leads to increased apoptosis of T-ALL cells [73].

### 4.5. Diffuse Large B-Cell Lymphoma

XBP1 was activated in 28% of DLBCL cases, 48% of plasmablastic lymphomas, and 69% of plasmacytic neoplasms. Nuclear XBP1 expression in diffuse large B-cell lymphoma was correlated with poorer response to therapy and shorter overall survival in contrast to tumors with non-expressing XBP1 [72]. In diffuse large B-cell lymphoma germinal center B-cell-like subtype (GCB-DLBCL), IRE1 expression is lower than in diffuse large B-cell-lymphoma-activated B cell (ABC-DLBCL). Thus, it can be concluded that IRE1-XBP1 downregulation distinguishes GCB-DLBCL from other DLBCL subtypes. Moreover, in GCB-DLBCL, the IRE1 expression is reduced to levels that prevent XBP1 activation. Furthermore, restoration of the IRE1 signaling pathway, through the expression of an active form of XBP1, inhibited GCB-DLBCL tumor growth in a mouse xenograft model. This indicates that, in contrast to its tumor-growth-promoting role in MM, IRE1/XBP1s activity may negatively impact tumor growth in GCB-DLBCL [74]. Hypoxia increases the expression of IRE1α and XBP1s in ABC-DLBCL and GCB-DLBCL cells, and higher expression is induced in ABC-DLBCL cells than in GCB-DLBCL cells [75]. In Ibrutinib-resistant DLBCL ABC-DLBCL lymphoma line (OCI-ly10-IR), it was found that ibrutinib-resistant cells showed markedly lower expression of UPR response marker genes, including XBP1s. Overexpression of XBP1s significantly enhanced ibrutinib-induced apoptosis in both sensitive and resistant cells. Importantly, ibrutinib was shown to induce UPR signaling in sensitive cell lines but not in DLBCL-resistant cell lines [76].

### 4.6. Other Lymphomas

Expression of IRE1α and XBP1s is increased in primary central nervous system lymphoma (PCNSL) with aggregative perivascular tumor-cell growth pattern (APVT) [75].

The IRE1α–XBP1 pathway is significantly upregulated in BL. Overexpression of c-Myc in BL leads to ER stress and increased IRE1α–XBP1 levels. Moreover, c-Myc overexpression induces BL growth and progression. The IRE1α–XBP1 pathway is important in maintaining ER homeostasis and preventing Myc-induced cytotoxic ER stress. These findings suggest that inhibition of the IRE1α–XBP1 axis in BL with current Myc overexpression may be a novel therapeutic target [64]. Additionally, conversely, induction of XBP1s overexpression in BL cells in vivo via transfection with a plasmid containing XBP1s-GFP resulted in rapid cancer cell death [77].

Primary effusion lymphoma (PEL) is associated with Kaposi’s sarcoma-associated herpesvirus (KSHV) infection. KSHV-infected PEL cells show reduced expression of IRE1α and XBP1s, compared with KSHV-uninfected PEL cells [78]. The IRE1α–XBP1 axis is also needed for the survival of this cancer type, as inhibition of the axis led to induction of apoptosis in PEL cells. These results suggest that inhibition of the IRE1α–XBP1 axis may be a novel therapeutic target in PEL [79].

Inhibition of the IRE1α–XBP1 pathway in NK cells obtained from HL patients impairs immune synapse formation between NK and Hodgkin/Reed–Sternberg cells. The inhibition also impairs NK morphology, motility, and migration in tested cells. Furthermore, the release of IFNγ and TNFα, like CD107a degranulation, is also dysfunctional. Interestingly, there was no sign found of activation of the IRE1α–XBP1 pathway in CD56brightCD16- NK cells from HL patients exposed to pembrolizumab [80].

### 4.7. Multiple Myeloma

**Figure 2:**
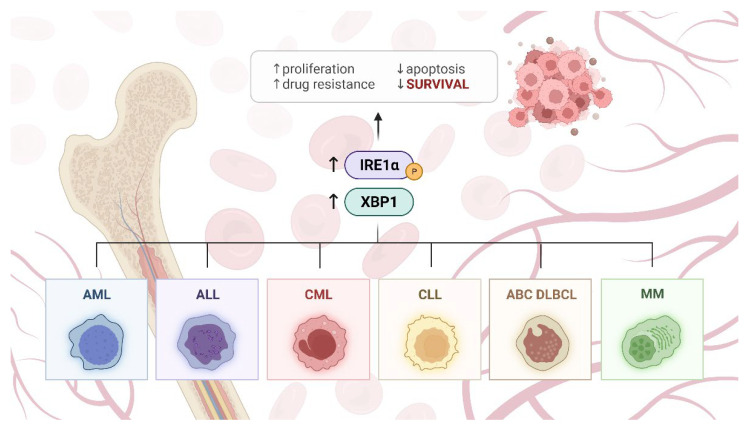
IRE1α-dependent signaling in blood cancer cells: The increased expression of IRE1α and its major substrate XBP1 may be observed in various hematological malignancies, including AML, ALL, CML, CLL, ABC DLBCL, or MM. In most cases, activation of IRE1α–XBP1 pathway results in enhanced proliferation and chemoresistance of cancer cells, while the rate of apoptosis is decreased, and patient survival is significantly poorer, compared with the mentioned tumor-non-expressing UPR-related proteins.

IRE1α–XBP1-dependent UPR branch activation is associated with many types of malignancies, including MM. MM cells possess a substantially dysregulated expression of XBP1 and IRE1α [81]. The level of IRE1α and XBP1 is often elevated in MM cases [82], which is directly linked to ER stress [83]. Furthermore, MM cell growth is apparently dependent on the IRE1α–XBP1 pathway [84]. While MM cell differentiation requires a moderate level of activity of UPR, XBP1 plays a crucial role in MM cell differentiation and maturation [85]. IRE1α is a possible factor that promotes osteoclastogenesis in MM [86]. On the other hand, IRE1α is also involved in proteasome-inhibitor-induced osteoblastogenesis in MM [16]. Poor response to bortezomib is associated with low basal XBP1s levels in MM cells [87]. Consistent with this finding, bortezomib-resistant cells are known to have decreased expression of the *Ern1* gene [88]. However, other studies describe that the change in XBP1s expression is a potential marker of response to bortezomib in MM cells rather than a cause of chemoresistance [89]. Further, BLOC1S1, a specific target of RIDD, is cleaved specifically by IRE1 in MM, but this cleavage does not affect MM cell viability under acute stress conditions [90]. For these reasons, IRE1α–XBP1s pathway was suggested to be a therapeutically useful vulnerability in MM [91,92] (Table 1, Figure 2).

## 5. Potential Application of IRE1α Inhibitors in Blood Malignancies

Depending on the mechanism of action and binding sites, the specific inhibitors of IRE1α activity may be divided into kinase inhibitors (type I and II) and RNase inhibitors [93]. Sunitinib, which is an FDA-approved anticancer drug and a type I inhibitor of the IRE1α kinase domain, effectively diminished splicing of XBP1 mRNA in H929 and U266 MM cell lines treated with an ER-stress activator tunicamycin. The mechanism involved inhibition of IRE1α autophosphorylation, which, in turn, affected the activity of the RNase domain and the unconventional splicing reaction [94]. Of type II kinase inhibitors of IRE1α, two compounds were selected for their potential utility in the therapy of blood cancers: N-{4-[(3-{2-[(trans-4-aminocyclohexyl)amino]pyrimidin-4-yl}yridine-2-yl)oxy]-3-methylnaphthalen-1-yl}-2-chlorobenzenesulfonamide (16) and (S)-2-chloro-N-(6-methyl-5-((3-(2-(piperidin-3-ylamino)pyrimidin-4-yl)yridine-2-yl)oxy)naphthalen-1-yl) benzenesulfonamide (18/KIRA8). KIRA8 markedly reduced the viability of MM and B-derived, non-myeloma cancer cell lines in 3D culture settings, in contrast to 2D cultures of these cell lines that demonstrate significantly lower levels of IRE1α–XBP1s [84]. This could also provide an explanation for the fact that the two compounds have previously proven to be ineffective as regards cell viability in the screening of the panel of over 300 native tumor cell lines, which included 15 MM cell lines [95]. Besides perturbations in IRE1α signaling, the expression of ERAD components, as well as secretion of Ig light chains, cytokines, and chemokines essential to MM growth, was also downregulated. The inhibitor also affected the growth of MM tumors in subcutaneous or orthometastatic mouse models and enhanced the efficacy of bortezomib and lenalidomide. Importantly, upon treatment with KIRA8, the function of non-malignant cells abundant in IRE1α–XBP1s (plasma cells, primary hepatocytes, pancreatic microislets) was preserved, and the drug was well-tolerated in treated animals. In patient-derived MM cells, KIRA8 attenuated the viability of CD138+ tumor cells while sparing either CD138− or CD138+ non-malignant cells. The effect was regardless of the derivation of cells from newly diagnosed or post-treatment-relapsed cases [84].

RNase inhibitor 4μ8C was shown to reduce XBP1 splicing and RIDD functionalities of IRE1α by blocking substrate access to the active site of the enzyme. Surprisingly, although the inhibitor attenuated the growth of MM cell lines, it did not induce acute toxicity in treated cells, nor did it exert a synergistic effect upon treatment with bortezomib. Thus, it was suggested that selective inhibition of RNase activity of IRE1α interferes with protein secretion and ER capacity rather than sensitizes cells to the effects of acute ER stress. As 4μ8C was found to be not suited for systemic administration, it may only be considered as a locally acting agent [81].

Toyocamycin, an agent derived from the *Actinomycete* strain, was able to prevent IRE1α-dependent XBP1 mRNA cleavage in vitro without interfering with IRE1α phosphorylation. In MM cell lines and primary samples obtained from patients, toyocamycin inhibited either ER-stress-induced or constitutive XBP1 expression. It also managed to overcome resistance to bortezomib in MM cells, even at nanomolar levels, and reduced the growth of MM xenografts in vivo [96]. Toyocamycin was also found to induce cytotoxicity against AML cells [13].

The MKC-8866 IRE1α inhibitor was tested in Philadelphia-positive (Ph+) ALL cells simultaneously with tyrosine kinase inhibitor (TKI) nilotinib. The combination of the two drugs (nilotinib at 0.5 µM and MKC-8866 at 30 µM) exerted a synergistic effect on cell viability, and this was additionally confirmed at the genetic level. The effectiveness of this dual inhibition was found to result from enhanced activation of the p38 MAPK and JNK pathway, as the addition of specific p38 and JNK inhibitors hindered the nilotinib- and MKC-8866-induced cytotoxicity [97]. Moreover, in SUP-B15 and TOM-1 cell lines, the dual therapy vastly potentiated the cytotoxic effect of dexamethasone, which is related to a possible regulation of glucocorticoid receptor (GR) signaling [98].

Another representative of the MKC family, MKC-3946, proved effective in the AML cellular model [13] and MM. Although treatment of MM cells with MKC-3946 alone showed modest growth inhibition and little toxicity, the compound demonstrated synergistic effects in combination with bortezomib or 17-AAG. It was observed that MKC-3946 blocked XBP1 splicing induced by chemotherapeutic agents and also enhanced apoptosis in CHOP-dependent mechanisms. Moreover, MKC-3946 significantly inhibited tumor formation in vivo in the MM xenograft model, and it was not toxic to normal mononuclear cells [99].

Recent high-throughput screening and topological data analysis have identified several *N*-acridine-9-yl-*N*’,*N*’-dimethylpropane-1,3-diamine (DAPA) analogs, among which *N*(9)-(3-(dimethylamino)propyl)-*N*(3),*N*(3),*N*(6),*N*(6)-tetramethylacridine-3,6,9-triamine (3,6-DMAD) was characterized by the most potent inhibitory action toward the IRE1α–XBP1 pathway. In contrast to the other analogs, 3,6-DMAD was found to act in a unique manner, which involved inhibition of both IRE1α oligomerization and RNase activity. The 3,6-DMAD-mediated inhibition of XBP1 splicing was cytotoxic to MM cell lines in vitro, and it affected the growth of MM tumor xenografts [100].

IRE1 inhibitor STF-083010 was tested in mice bearing human MM xenografts with great efficacy, as observed by significant tumor growth inhibition [101,102,103]. The cytostatic and cytotoxic effect of STF-083010 was dose- and time-dependent. In an ex vivo experiment, STF-083010 was selectively cytotoxic to CD138+ cells isolated from MM patients, compared with control cells obtained from healthy donors [101,104]. A106 moiety (2-hydroxy-1-naphthaldehyde; HNA) is a product of STF-083010 spontaneous hydrolysis with retained, full RNase inhibitory activity. In patient-derived pre-B ALL cells, either STF-083010 or HNA affected cell proliferation and survival, resulting from G0/G1 cell-cycle arrest, and the effect was dose-dependent. It is worth noting that STF-083010 treatment is also effective against refractory ALL with the BCR-ABL1T315I mutation. Moreover, pre-B ALL and Ph^+^ ALL cells (including the multi-drug-resistant Ph+ ALL phenotype carrying mutant BCR-ABL1T315I) were significantly more sensitive to treatment than mature B-cell lymphoma or MM cells. Further, treatment with HNA significantly prolonged survival in xenotransplant recipients, pre-injected with patient-derived pre-B ALL cells at the low count (50,000 and 10,000). This provides a rationale for the potential utility of the compound in the prevention of relapse resulting from a small number of drug-resistant cells that may reinitiate leukemia [14]. Moreover, STF-083010 was found to attenuate XBP1 splicing and exhibit significant cytotoxicity in AML cells [13]. In the same study, HNA also blocked XBP1 mRNA splicing in AML cells with subsequent induction of cytotoxicity, and the effect was synergistic upon the addition of bortezomib or As_2_O_3_. It was suggested that the toxic effect was associated with an increase in p-JNK levels and a decrease in p-phosphoinositide 3-kinase (p-PI3K) and p-MAPK levels. Inhibition of IRE1α resulted in caspase-dependent apoptosis and cell-cycle arrest at the G1 phase and increased expression of miR-34a that conferred cellular resistance to HNA [13]. Interestingly, murine bone marrow cells with deleted XBP1 were resistant to compound-induced growth inhibition.

A novel, highly selective RNase inhibitor, B-I09, mimicked XBP1 deficiency in CLL cells by upregulating IRE1a expression level and compromising BCR signaling. The agent also suppressed leukemic progression in CLL tumor-bearing mice without inducing systemic toxicity and synergized with ibrutinib to induce apoptosis in MM, lymphoma, and mature B-cell leukemia cells [65]. In c-Myc–overexpressing BL, B-I09-treated cells displayed a dose-dependent decrease in XBP1s protein level that correlated with cell proliferation and viability. The effect of B-I09 was more prominent in P493 high-Myc cells, whereas in low-Myc- or no-Myc-expressing cells, it was more subtle. Moreover, the pro-apoptotic effect of B-I09 was significantly higher than that of traditional chemotherapeutics, doxorubicin, or JQ1, and together with these drugs, B-I09 exhibited a synergistic effect. Among the three tested CLL cell lines (MEC1, MEC2, and WaC3), each with different c-Myc levels, WaC3 cells were the most sensitive to the growth arrest and apoptosis induced by IRE1α inhibitors B-I09 and 4μ8C, even though they grew the most slowly. B-I09 also inhibited the growth of P493 high-Myc xenograft without inducing systemic toxicity, and 8498 cells isolated from the Eκ/Myc mouse lymphoma model were also sensitive to B-I09 treatment [64] (Table 2).

In contrast to the specific inhibitors of IRE1α activity, the mechanism of action of several proteasome inhibitors (PIs) such as bortezomib is based on an accumulation of misfolded proteins in the ER and induction of ER stress. PIs can inhibit the splicing of XBP1 by suppressing the RNase domain of IRE1 and also reduce the generation of XBP1s by enhancing the stability of XBP1u proteins. Such XBP1s deficiency makes MM cells susceptible to ER-stress-mediated apoptosis. Further, PIs rapidly induce components of the terminal UPR, including the PERK-dependent, pro-apoptotic ATF4–CHOP pathway, which also leads to MM cell death. As PIs promote monoclonal Ig-induced ER stress and related death in MM cells, PIs can be an effective treatment for MM [108,109]. CB-5083, a novel, orally available inhibitor of p97, a central component in the ubiquitin–proteasome system, was shown to significantly reduce the viability of 10 human B-ALL cell lines. The mechanism involved activation of ER stress, as evidenced by overexpression of specific chaperones, IRE1α–XBP1s, and PERK–CHOP branches of the UPR. Interestingly, the absence of XBP1 increased cell sensitivity to CB-5083, suggesting that the activity of CB-5083 is counteracted by XBP1 splicing, probably by mitigating ER stress [110].

The cyclin-dependent kinase (CDK) inhibitor SCH727965 (dinaciclib) was also found to diminish XBP1s and GRP78 activity and induce cell death in human leukemia and MM cells treated with thapsigargin or tunicamycin, even at extremely low concentrations. In contrast to the typical IRE1α RNase inhibitors, dinaciclib attenuated nuclear localization and accumulation of XBP1s rather than its transcription, translation, or splicing. It was suggested that this effect could be secondary to CDK1/5 inhibition. Consistent with these findings, dinaciclib downregulated XBP1s expression and inhibited MM cell growth in vivo [111]. In line with all mentioned findings, the KIRA8 compound seems to be a promising candidate for MM treatment, as it was found to selectively target MM while preserving the function of non-cancerous secretory cells intact. Other relevant results for the development of targeted strategies reveal that multi-drug-resistant Ph+ ALL harboring BCR-ABL1T315I is sensitive to STF-083010 treatment and that B-I09 was exceptionally effective against BL cells with high levels of Myc. Further, the inhibitory compounds such as KIRA8, toyocamycin, or MKC-3946 potentiated the cytotoxic effect of commonly used anticancer drugs bortezomib, imatinib, or ibrutinib, which provides the basis for the development of novel combination therapies that could bypass chemoresistance. It should also be considered to target XBP1 indirectly, as an auxiliary pathway that potentiates the drug effect rather than the main target, as in the case of CB-5083 or dinaciclib.

As it is known that IRE1α indirectly activates various proteins, including BCL2, it seems possible that inhibitors of BCL2 family members may exhibit synergism with IRE1α inhibitors in blood cancer treatment. The BCL2 inhibitor venetoclax was already approved for use in the treatment of CLL [112]. However, to date, there are no studies to confirm such potential synergism.

In terms of DLBCL, there are also new exciting therapeutic approaches currently under investigation [113]. Some of the drug targets such as PI3K/Akt are known to interact with IRE1α signaling; thus, it is worth investigating whether the combination of inhibitors of these pathways would potentiate the antiproliferative and pro-apoptotic effect in DLBCL cells. Novel IRE1α inhibitor B-I09, when co-administered with BTK inhibitor ibrutinib, orchestrated apoptosis in several hematopoietic malignant cell lines, but little is known about the effect of the two drugs in DLBCL [65].

## 6. Conclusions

Blood cancers, which comprise several types of leukemia, lymphomas, and myeloma, can develop from various types of blood cells at any level of their differentiation, and thus, they constitute a highly heterogeneous group of neoplasms. Dysregulation of IRE1α can cause many different diseases including blood malignancies because IRE1α acts directly and indirectly through downstream pathways on many important molecular regulatory mechanisms in the cell. IRE1α interacts directly with the cell through, among other things, RIDD by regulating the DNA damage response, affecting DNA repair, cell-cycle arrest, and apoptosis. In comparison, the indirect interaction occurs via XBP1, affecting the production of important proteins such as Il-6, upregulation of Myc proto-oncogene, or plasma cell differentiation. Therefore, in a number of hematological malignancies, such as CLL, AML, pre-B ALL, and DLBCL, the IRE1α–XBP1 branch is significantly activated. This is probably due to prolonged ER stress conditions or enhanced secretory capacity in the ER, which are prominent in highly proliferating blood cancer cells. Especially, malignantly transformed plasma cells in MM possess a robust secretory apparatus, essential for Ig hyperproduction in the ER [84]. The IRE1α–XBP1 pathway was found to regulate the differentiation of plasma cells, but when misregulated, it can also promote uncontrollable proliferation in MM [94]. Importantly, IRE1α does not play such a key role in maintaining proteostasis of differentiated secretory cells, which supports the idea of potential clinical application of IRE1 inhibitors that may have limited toxicity toward normal cells [81]. This theory led to the conclusion that pharmacological blockage of IRE1α RNase-dependent XBP1 splicing may be a potential new therapeutic option that selectively targets MM. In resistant phenotypes of DLBCL or MM, decreased levels of XBP1 can be observed, but it has not yet been established whether this is only the effect of exposure to the drug or a compensatory mechanism that induces resistance in treated cells. Similarly, some studies have reported contradictory results—for instance, it was found that increased expression of XBP1s in AML correlates with a more favorable clinical course and better prognosis. Then, it would be essential to validate these results in another cohort. Partial inhibition or activation of IRE1α should also be considered to restore its physiological levels and, more precisely, target cancer cells that already have dysregulated UPR signaling. Many promising compounds are still not fully investigated, and widely applied 2D culture models proved to have limitations and be unreliable in terms of translation. As most blood malignancies are characterized by poor prognosis, further extensive research regarding mentioned aspects and improvement of preclinical models could give new hope to many blood cancer patients.

## Figures and Tables

**Table 1:** Distinct roles of IRE1α-dependent XPB1 signaling in various hematological diseases.

Disease Name	The Role of XBP1
Chronic myelogenous leukemia (CML)	XBP1 promotes the survival of hematopoietic stem cells (HSCs) under ER stress [63].
Chronic lymphocytic leukemia (CLL)	Myc-overexpression-activated XBP1 sustains cell proliferation and viability [64].
	XBP1s supports cell growth and increases IgM production and BCR signaling [65].
Acute myeloid leukemia (AML)	XBP1s regulates AML cell survival [13,69] and expansion [69].
	Activation of XBP1 is associated with a more favorable course of the disease [67].
	XBP1 induction in the AML niche contributes to adaptive changes in stromal cells of the bone marrow [68].
Mast cell leukemia (MCL)	Splicing of XBP1 is crucial for cell proliferation and survival [70].
Pre-B acute lymphoblastic leukemia (ALL)	XBP1 is highly expressed in patients, induces cancer survival and proliferation, and is associated with poor outcomes [14].
Diffuse large B-cell lymphoma (DLBCL)	Activated XBP1s correlates with poorer clinical outcome and shorter overall survival [72,75] and is associated with more invasive phenotypes [75].
Activated B-cell (ABC) DLBCL	Lower XBP1 levels induce resistance to ibrutinib [76].
Germinal center B-cell–like (GCB) DLBCL	Downregulation of XBP1 is pro-survival and supports tumor growth/XBP1s activity and negatively impacts tumor growth [74].
Burkitt’s lymphoma (BL)	XBP1 splicing is enhanced in Myc-overexpressing cells and has a protective role [64].
	Overexpression of XBP1s is lethal to BL cells [77].
Primary effusion lymphoma (PEL)	Basal activation of XBP1 is essential for PEL cell survival, the release of cytokines, and autophagy regulation [79].
	Reduced basal splicing of XBP1 makes cells susceptible to ER-stress-induced apoptosis [78].
Multiple myeloma (MM)	XBP1s is highly expressed and has pro-survival effects on MM cells [83]; it is essential for MM growth, chemoresistance [84], differentiation, and maturation [85].
	XBP1s is a key regulator of osteoblast differentiation induced by proteasome inhibitors [16].
	Splicing of XBP1 is involved in MM-cell-derived small extracellular vesicle (EV)-induced osteoclast differentiation [86].
	High levels of XBP1 correlate with a better response to bortezomib [82].
	Low levels of XBP1s induce resistance to bortezomib [87].
	Change in XBP1 expression determines the effectiveness of bortezomib treatment [89].

**Table 2:** Comparison of different inhibitors of IRE1α protein domains by their mechanism of action and effectiveness in hematological diseases.

Name of the Inhibitor	Mechanism of Action	Study Model	First Scientific Evidence
Sunitinib	Type I kinase inhibitor	MM (H929 and U266 cells) [94]	[105]
KIRA8	Type II kinase inhibitor	MM and B-cell lymphoma cell lines [84]	[95]
4μ8C	RNase inhibitor	MM (MM1.R cells) [81]	[81]
Toyocamycin	RNase inhibitor	MM (cell lines, patient samples, mouse xenografts) [96], AML (patient samples) [13]	[96]
MKC-8866	RNase inhibitor	Ph^+^ ALL (SUP-B15 and TOM-1 cells, genetic mouse model) [97,98]	[106]
MKC-3946	RNase inhibitor	AML (patient samples) [13], MM (MM.1S and MM.1R cells) [99]	[99]
3,6-DMAD	Unknown	MM (RPMI 8226 and MM1.R cells and xenografts) [100]	[100]
STF-083010	RNase inhibitor	AML (patient samples) [13], pre-B ALL and Ph+ ALL (genetic and patient-derived xenografts) [14], MM (cell lines, xenografts) [101]	[101]
A106/HNA	RNase inhibitor	AML (patient samples) [13], pre-B ALL and Ph+ ALL (genetic and patient-derived xenografts) [14]	[107]
B-I09	RNase inhibitor	BL (human and mouse cells), CLL (human [64] and mouse cells [65]	[65]
